# Efficient Donor Impurities in ZnO Nanorods by Polyethylene Glycol for Enhanced Optical and Glutamate Sensing Properties

**DOI:** 10.3390/s16020222

**Published:** 2016-02-06

**Authors:** Sami Elhag, Kimleang Khun, Volodymyr Khranovskyy, Xianjie Liu, Magnus Willander, Omer Nur

**Affiliations:** 1 Department of Science and Technology, Campus Norrkoping, Linkoping University, SE-60174 Norrkoping, Sweden; kimleang.khun@liu.se (K.K.); magnus.willander@liu.se (M.W.); omer.nour@liu.se (O.N.); 2 Department of Physics, Chemistry and Biology (IFM), Linköping University, 58183 Linköping, Sweden; volodymyr.khranovskyy@liu.se (V.K.); xianjie.liu@liu.se (X.L.)

**Keywords:** glutamate, ZnO nanorods, doping, potentiometric sensor

## Abstract

In this paper, we show that the possibility of using polyethylene glycol (EG) as a hydrogen source and it is used to assist the hydrothermal synthesis of ZnO nanorods (ZNRs). EG doping in ZNRs has been found to significantly improve their optical and chemical sensing characteristics toward glutamate. The EG was found to have no role on the structural properties of the ZNRs. However, the x-ray photoelectron spectroscopy (XPS) suggests that the EG could induce donor impurities effect in ZnO. Photoluminescence (PL) and UV-Vis. spectra demonstrated this doping effect. Mott-Schottky analysis at the ZNRs/electrolyte interface was used to investigate the charge density for the doped ZNRs and showed comparable dependence on the used amount of EG. Moreover, the doped ZNRs were used in potentiometric measurements for glutamate for a range from 10^−6^ M to 10^−3^ M and the potential response of the sensor electrode was linear with a slope of 91.15 mV/decade. The wide range and high sensitivity of the modified ZNRs based glutamate biosensor is attributed to the doping effect on the ZNRs that is dictated by the EG along with the high surface area-to-volume ratio. The findings in the present study suggest new avenues to control the growth of n-ZnO nanostructures and enhance the performance of their sensing devices.

## 1. Introduction

L-glutamate acid (Glu) is an important amino acid widely used as a food additive because of its taste enhancing property. In neurochemistry, it is a major excitatory neurotransmitter of the vertebrate central nervous system [1,2]. It is reported that abnormal concentrations of Glu may indicate disorder such as trauma, stroke, epilepsy and hypoglycemia [1]. Therefore, sensitive detection of Glu in a clinical laboratory is very important and is also of interest in evaluating the quality of food. Commonly, L-glutamate oxidases (GluOx) catalyze the oxidation of L-glutamic acid to 2-oxoglutaric acid, simultaneously generating NH_3_ and H_2_O_2_. However, H_2_O_2_ charge transfer kinetics is slow on most of conductive substrates in addition the L-glutamic acid is a poor substrate [3,4]. Thus, there has been a great interest in seeking a highly catalytic electrode material to detect Glu. Different methods have been developed to determine Glu, e.g., chromatographic [5] fluorescence [6], and electrochemical methods [7] among these analytical methods, the electrochemical methods are considered as an approach with the most potential. The potentiometric method as one in the electrochemical family has the advantages of simplicity, rapidity, and high sensitivity. However, still there are a few areas in which improvement is needed, such as lack of stability, high response time, low reproducibility and wide range of detection.

In addition to fast electron transfer kinetics between the enzyme and the electrode, the biocompatible electro-catalytic activities, accompanied with a high surface area to volume ratio of the nanostructured metal oxides, make them suitable for the development of third generation biosensors [8,9,10]. The aqueous chemical methods for the production of functional nanostructures [11] have opened a bright field not only for the control of the morphology of the nanostructures but also for doping research. It is well known that, the dopant nature can effectively modify the band gap of the semiconductor *i.e.*, can be decreased or increased [12,13]. Previously, we reported the use of different surfactants to assist the aqueous chemical growth of ZnO nanostructures, and have shown that the surfactants are able to function as a growth template to control the morphology and as a dopant to modify the optical and sensing properties [14].

**Scheme 1:**
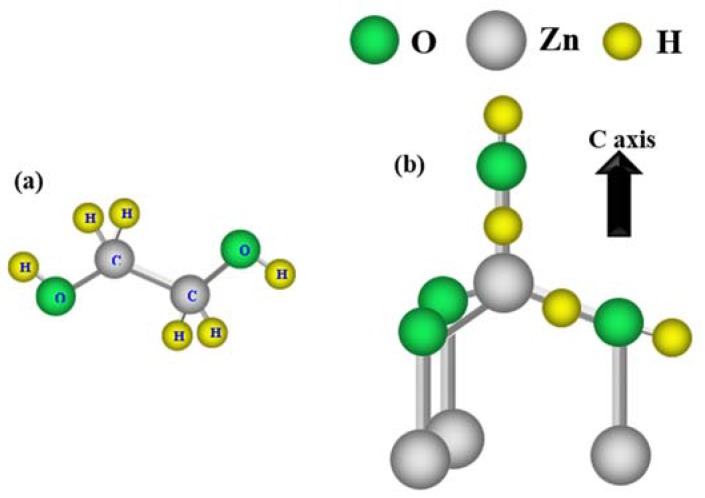
(**a**) Ethylene glycol molecule with abundant hydrogen, and (**b**) representation of the four H sites in ZnO according to [20].

The focus of the present paper is the use of ZnO nanorods (ZNRs) based modified electrodes to further increase the sensitivity towards Glu and improve the performance of the sensing electrode. In order to do that, we have systematically studied the influence of different concentrations of ethylene glycol (EG, Scheme 1a) added to act as a dopant source. The ZNRs have been synthesized by the standard hydrothermal method and the EG is used as a doping source. This is in contrast to the use of the EG as a growth template as reported by others before [15,16,17,18,19]. Here our aim is to modulate the electronic structure while keeping the morphology of the synthesized material as nanorods. The reason behind the morphology preservation is also discussed. The GluOx was immobilized on the ZNRs for the development of sensitive Glu biosensor using the potentiometric technique. The crystal morphology and electronic structure have been fully investigated. Further, the effect of the EG on Glu sensing was also highlighted.

## 2. Experimental Section

### 2.1. Chemicals

L-glutamic acid monosodium salt (98%), Streptomyces sp. L-glutamate oxidase (GluOx, EC 1.4.3.11), bovine serum albumin (BSA), phosphate buffered saline solution (PBS, pH = 7.3) and glutaraldehyde (50% wt. in water), zinc nitrate hexahydrate ZnNO_3_·6H_2_O, (ZNH) hexamethylenetetramine C_6_H_12_N_4_ (HMT), polyethylene glycol 2’000 C_2_H_6_O_2_ (EG), and zinc acetate dihydrate Zn(CH_3_COO)_2_·2H_2_O, methanol CH_3_OH, and potassium hydroxide KOH were all of analytical grade and were purchased from Sigma Aldrich Sweden and used without further purification.

### 2.2. Growth of the Modified ZNRs on Au Coated Glass

The growth of the ZNRs was performed using the hydrothermal method as reported in [14]. The growth solution used in this experiment was an equimolar concentration of 0.05 M of ZNH and HMT solution in de-ionized water with the presence of different concentrations of the EG (0, 0.05, 0.1 and 0.15% (w/v)). EG was used as the impurity that would yield a desired modulation into the electronic structures of the ZNRs. The substrates were fixed to a Teflon sample holder and then dipped into the growth solution with face downward and they were then placed in an oven for 6 to 7 h at 90 °C. After completing the growth duration, the samples were washed by de-ionized water and dried with blowing nitrogen gas.

### 2.3. Characterization

Field emission scanning electron microscopy (SEM) for morphological analysis was performed by a LEO 1550 Gemini field emission gun used at 5 kV. The crystal quality of the ZNRs was studied by X-ray powder diffraction (XRD) using a Phillips PW 1729 powder diffractometer equipped with CuKα radiation (λ = 1.5418 Å) using a generator voltage of 40 kV and a current of 40 mA. Surface and chemical composition analysis were investigated by X-ray photoelectron spectroscopy (XPS) technique and were measured by Scienta ESCA200 using a vacuum generators a monochromatic Al (Kα) radiation. In order to calculate the carrier density, electrochemical impedance spectroscopy (EIS) measurements were performed in a three-electrode cell using an SP-200 potentiostat (Bio-Logic, Claix, France), with a platinum sheet as a counter electrode and a standard Ag/AgCl in 3 M KCl as a reference electrode. The EIS was performed using the following parameters: amplitude of 20 mV; frequency range of 10 kHz–0.1 Hz, and a potential range: −0.8 V–(+0.8 V). The electrolyte used was 0.1 M Lithium perchlorate (LiClO_4_) in carbonate propylene to avoid ZNRs decomposition [21].

### 2.4. Immobilization of ZNRs with GluOx and Electrochemical Measurement

A 20 µL of GluOx solution was added to 200 µL (0.01 M PBS). 2.5 mg of BSA used as an enzyme stabilizer and 10 µL of glutaraldehyde solution used as the cross linker [22] were added to this solution and immobilized on the ZNRs through electrostatic physical adsorption method. After that all the electrodes were left to dry in a fume hood at room temperature for one night. All the functionalized biosensor electrodes were kept in a dry Petri-plate at 4 °C when not in use. Before the insertion of GluOx immobilized sensor electrode in a Glu solution, it was soaked in PBS in order to achieve a stable response as well as to remove the extra molecules of GluOx on the surface of the electrode. A stock solution of 10 mM of Glu was prepared in few drops of 0.01 M HCl [23] and finally mixed with 0.01 M PBS of pH 7.3. Low concentrations of Glu were obtained by dilution. All the electrochemical measurements were performed using a Keithley 2400 model at room temperature.

## 3. Results and Discussion

### 3.1. Morphology and Structural Analysis

**Figure 1:**
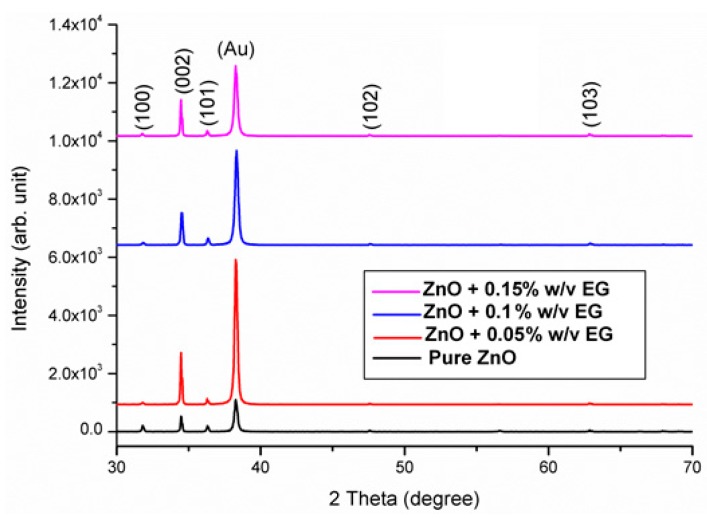
XRD of undoped ZNRs and ZNRs with EG, and all of them indicating a c-axis oriented structure.

XRD spectra of the grown ZNRs with and without the EG are shown in Figure 1. The measurement acquisition parameters were the same when collecting the data. All samples show similar XRD patterns. All the ZNRs grown on Au substrates show a preferred (002) growth orientation, indicating that the ZNRs grow along the *c*-axis direction and many diffraction peaks can be indexed to the hexagonal wurtzite ZnO (JCPD NO 36-1451).

**Figure 2:**
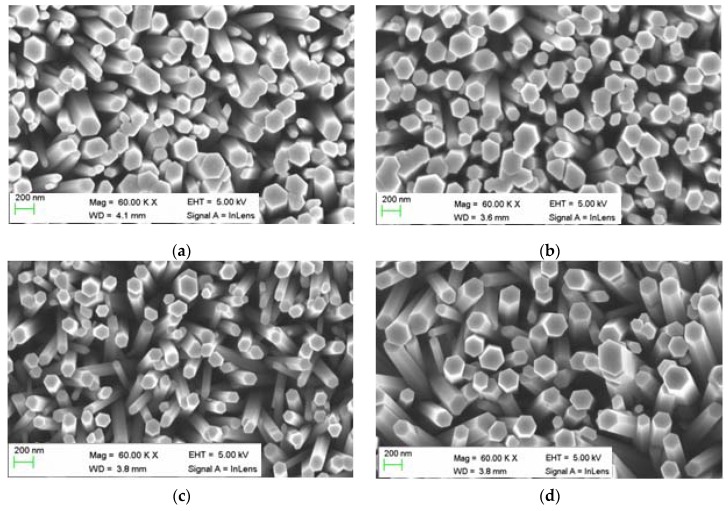
SEM of (**a**) pristine and doped (**b**) with 0.05, (**c**) 0.1, (**d**) 0.15% (w/v) of EG.

Figure 2a–c shows the SEM images of the ZNRs obtained for different EG concentrations. All the ZNRs were dense, vertically aligned, and with a diameter in the range of 200–400 nm. However, ZNRs doped with 0.1% (w/v) of EG exhibited low density distributions (Figure 2c). In the experiment, water-equimolar zinc nitrate hexahydrate and HMT of 0.05 M were mixed with different concentrations of EG. However, as the EG concentration is increased from 0 to 0.05, 0.1 or 0.15% (w/v) of EG solution, the morphology is observed to be preserved as ZNRs that are usually grown when using HMT as a buffer medium [14]. The predicted ZNRs formation mechanism with the use of HMT can be described as:

(CH_2_)_6_N_4_ + 6H_2_O → 6COH_2_ + 4NH_3_ (1)

NH_3_ + H_2_O → NH_4_^+^ + OH^−^ (2)

2OH^−^ + Zn^2+^ → Zn(OH)_2_ → ZnO (s) + H_2_O (3)

The HMT acts as a buffer medium during the growth and supplies ammonia (NH_3_) which in turn undergoes hydrolysis and generates hydroxide ions (OH^−^) and finally OH^−^ ions react with Zn^2+^ ion and yields Zn(OH)_2_. Zn(OH)_2_ is playing an important role in the growth by deciding the pH value and crystal quality, therefore could be referred as growth unit [24]. Hence, the ZnO nanostructures morphology can be well controlled through Zn(OH)_2_ unit [14]. Many researchers have also incorporated organic additives to the ZnO material, and their exclusive results were demonstrated by introducing new morphology. Accordingly, some characteristics e.g., optical, electrical, and catalytic *etc*. have been modulated [16,17]. In contrast, the incorporation of inorganic material into ZNRs with a certain concentration has no role on the morphology, but the aforementioned characteristics were notably modified [25,26]. Note, the EG is completely miscible with water and has found many applications in current technology as antifreeze when mixed with water [27]. This is probably due to its strong hydrogen bonding interaction in water [28]. Therefore, we believe that the disruption of hydrogen bonding when dissolved in water provides a rich hydrogenated-environment for the growth of ZNRs. It is important also to note that hydrogen is relatively very small in volume and the possibility of the incorporation inside the ZNRs structure is highly expected. Limpijumnong *et al.*, and E.V. Lavrov [20,29] have investigated the configuration of hydrogen when incorporated into ZnO under hydrostatic pressure and they reported that hydrogen has four possible locations to reside in ZnO, *i.e.*, it might be a shallow donor or might not be a shallow donor (Scheme 1b). Lavrov *et al.*, have also reported that hydrogen-related shallow donor in ZnO were studied by photoconductivity and infrared absorption spectroscopy [30]. In the present work, and from the XRD and SEM results, the EG has showed no influence on the structure and morphology of ZnO. This outcome is presumably attributed to the fact that the EG is completely miscible in water and as a result, the water-EG solution would act as an impurity source. In order to investigate the incorporation of the EG more closely we have attempted to grow ZNRs with the absence of HMT, *i.e.*, zinc nitrate hexahydrate as Zn source and EG as precursor but could not get any success in the growth of any ZNRs.

**Figure 3:**
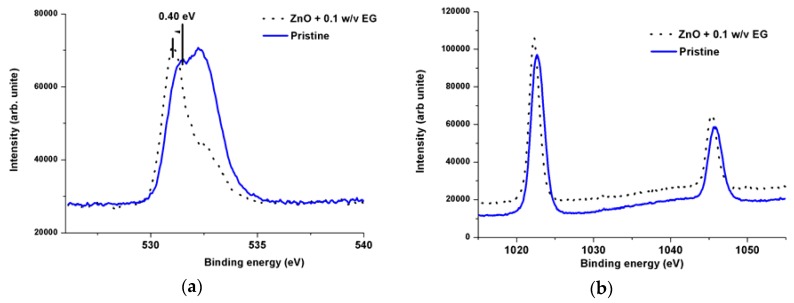
XPS study of the as grown ZNRs pristine (solid line) and doped with 0.1% w/v EG on Au (**a**) O 1s and, (**b**) Zn 2p spectra.

Oxygen 1s (O 1s) and zinc 2p (Zn 2p) XPS spectra of the product (pristine and 0.1% (w/v) EG doped ZNRS) are shown Figure 3a,b, where the spin-orbital splitting peaks at ∼1022.501 and ∼1045.820 eV, are assigned with the Zn 2p_3/2_ and Zn 2p_1/2_, respectively [31]. A strong pronounced peak in the case of the ZNRs grown without EG at ∼532 eV is clearly observed (Figure 3a) and it belongs to the hydroxyl group (OH) and adsorbed water [32]. While for the ZNRs grown with the addition of 0.1% (w/v) EG, this peak appears as a weak distinct shoulder, indicating that no OH removal has taken place. Van de Walle [33], suggested that the OH bonding could be incorporated inside the ZnO structure *i.e.*, the addition of a proton turning the oxygen into an element behaving much like fluorine and hence appears as a weak shoulder compared to the case of the ZNRs grown without EG. Furthermore, the presence of the EG, has caused the core level of the O 1s to be shifted by ∼ 0.4 eV to lower binding energy (see Figure 3a) It is well established that small concentrations of native point defects and impurities can significantly affect the electrical and optical properties of semiconductors [13]. Hence, due to this, n-type conductivity has been increased.

**Figure 4:**
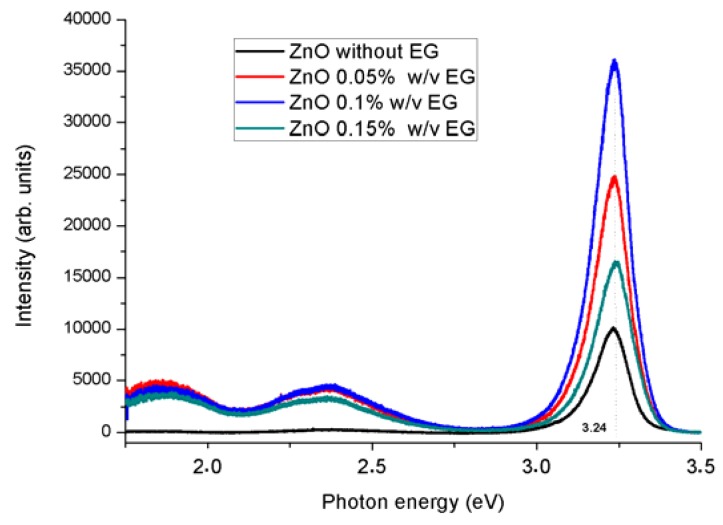
PL spectra of ZNRs grown on Au with and without presences of EG showing a sharp UV peak accompanied by two broad visible emission peaks.

### 3.2. Optical Characteristics

Figure 4 show the PL spectra of the ZNRs taken for different amounts of EG. The intrinsic optical transitions between the electrons in the conduction band and the holes in the valence band “UV photoluminescence”, in addition to the defect-related transitions “visible emission” can both clearly be seen [34]. The photoluminescence intensity (UV in this case) is explicitly increased. This suggests that the doping is due to the hydrogen incorporation. However, this dependence is observed, except of the cases with EG exceeding 0.1% (w/v). This observation is also in agreement with C Tsiarapas *et al.*, [35]. They correlated the best results for Schottky diodes to a certain amount (33.3%) of hydrogen. Therefore, one can infer that, a low amount of EG introduces hydrogen which acts as impurities and lead to increase the passivation of nonradioactive defect [36,37,38].

**Figure 5:**
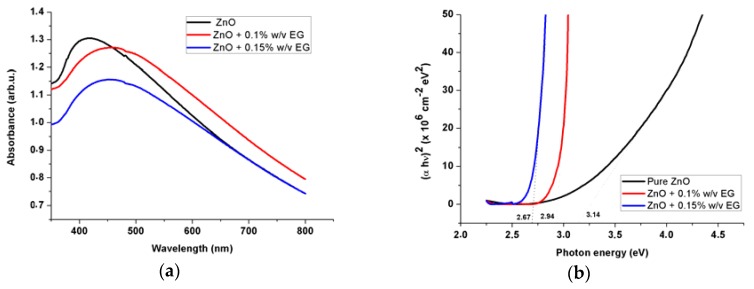
UV–Vis absorption spectra, shows the plot of (αE)^2^ *versus* photon energy for the ZNRs grown with different amount of EG.

Figure 5a show that all the absorbance spectra for the ZNRs grown with various amounts of EG exhibited spectral features and are very similar to those reported for pure ZnO. The absorption peaks for the ZNRs grown with 0, 0.1, 0.15% (w/v) of the EG was observed at ∼2.82, 2.66 and 2.62 eV, respectively. It can be seen that the absorption edge moves towards a higher-wavelength with increased EG concentration. The optical band gap can be evaluated by using Tauc model: $\left(\alpha h\nu \right)=A{\left(h\nu -E_{g}\right)}^{1/2}$ [39], where *E*_g_ is the band gap, and α is the absorption coefficient (cm^−1^) calculated from $x=e^{-\alpha d}$ [40], where x(λ) is the absorbance, and d is the length of ZNRs (∼1 µm). Next, we plotted (αhν)^2^ *versus* E in Figure 5b for all three ZNRs. The linear extrapolation of (αhν)^2^ gives the band gap values of each ZNRs. For the pristine ZNRs the obtained value was ∼3.16 eV, while for the NRs grown with addition of the EG, the corresponding values were ∼2.95, and 2.67 eV for NRs grown using 0.1, and 0.15% (w/v) EG, respectively. It is also worth mentioning that, in semiconductor nanocrystals; the dimension of the nanostructures, impurities and defects concentration can affect the physics of the band gap [13,41]. That means the band gap of bulk materials can be larger when compared with nanomaterial forms and can be decreased or increased depending on the dopant nature. Therefore, the decrease in the optical band gap as the EG concentration is increased could be ascribed to the EG impurities that could act as shallow donors into the band gap of ZnO.

### 3.3. Mott-Schottky Analysis

**Figure 6:**
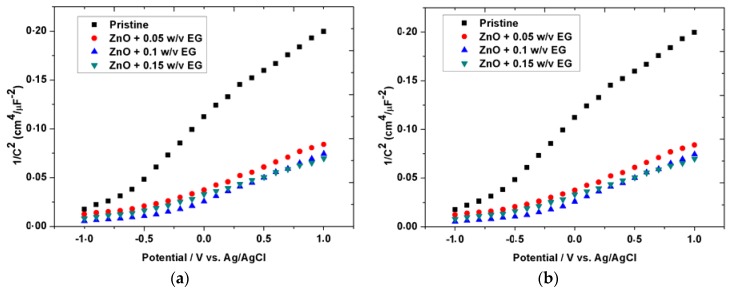
(**a**) & (**b**) Mott-Schottky plots of the ZNRs grown on Au with different amount of EG at 5 kHz in 0.1 M LiClO_4_ and in (**b**) shows the increases in capacitances upon the increase of the EG amount.

Moreover, an analysis at ZNRs/electrolyte interface was performed using the Mott-Schottky method to connect the effect of EG to ZNRs’ carrier concentrations. Figure 6a–b compares the capacitances values obtained for the bare ZNRs with that grown with 0.05, 0.1, 0.15% (w/v) of EG. The Mott-Schottky plot of $1/C^{2}$ = $\frac{2}{N_{d}e\varepsilon_{s}}\left[\left(V-V_{fb}\right)-kT/e\right]$ *versus* the voltage (Figure 6a) is used to extract the doping density (N_d_) from the slope of the straight line and flat band voltage (V_fb_) is obtained from the intercept [42]. Where C is the capacitance, V is the electrode potential, K is the Boltzmann constant, T is the temperature, e is the electron charge, and $\varepsilon_{s}=10$ is the dielectric constant of ZnO [20]. The extracted values for the N_d_ and the V_fb_ for all the ZNRs samples at a selected frequency (∼ 1.5 kHz) are summarized in Table 1. As the negative potential increased more electrons are repelled from the ZNRs surface and the depletion region widens, thereby increasing the space charge capacitance [43]. From Figure 6b, it can be seen that the capacitances increase with the increase of the EG amount. This capacitance reached a value of ∼ 200 C^2^ µF^2^/Cm^4^ for an amount of 0.1% (w/v) of the EG as compared to the pristine sample that showed a maximum capacitance of ∼60 C^2^ µF^2^/Cm^4^. Note that, upon adding 0.15% (w/v) of EG, is also accompanied with an increase in the capacitance (from 57 to 124 C^2^ µF^2^/Cm^4^). This means that the EG is acting as an impurity, nevertheless, we observed that at an amount of 1.5% (w/v) EG the effect is lower than for 0.1% (w/v), which means that a reversed effect is observed. According to Table 1, the free carrier concentration for the 1.5% (w/v) EG sample is less than for the 0.1% (w/v). This is can be interpreted in terms of the self-compensation mechanism [12], which is commonly observed for II–VI semiconductors and it has been interpreted as a recombination of free carriers from dopants with oppositely charged compensating native point defects (vacancies, interstitials. *etc*.).

**Table 1:** Influence of EG doping on flat band voltage (V_fb_) and the doping density (N_d_) of ZNRs.

ZNRs	V_fb_ (V)	N_d_ (cm^−3^)
Pristine	−1.09	2.81 × 10^19^
+0.05% (w/v) EG	−1.1	5.37 × 10^19^
+0.1% (w/v) EG	−0.87	1.39 × 10^20^
+0.15% (w/v) EG	−0.82	7.58 × 10^19^

### 3.4. The Detection of Glu by Modified ZNRs

**Scheme 2:**
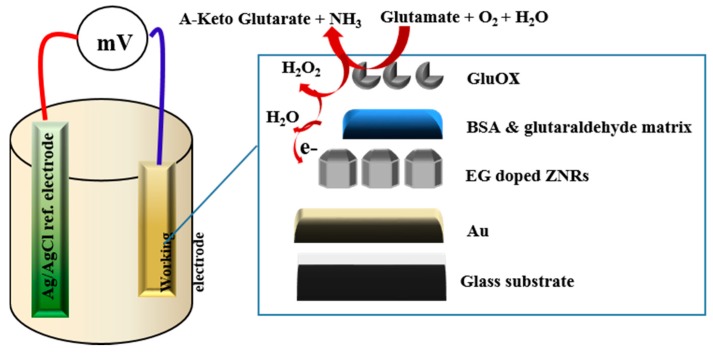
Represents the potentiometric measurements; working electrode and charge environment of a proposed biosensor.

**Figure 7:**
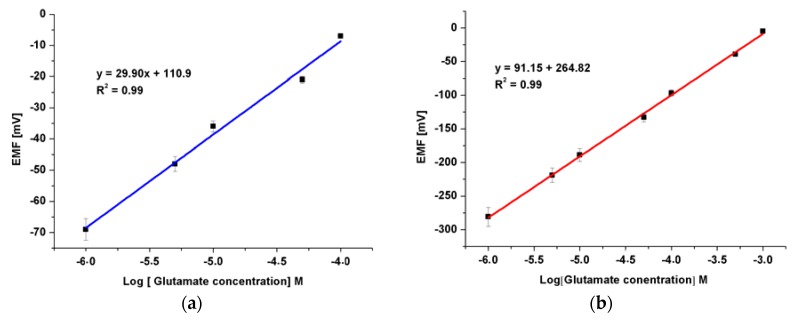
The calibration curve of the fabricated Glu biosensor based on ZNRs grown (**a**) without EG and (**b**) with 0.1%(w/v) of EG , more than six sensor electrodes have been investigated using the same set of conditions and the same functionalized membrane.

**Figure 8:**
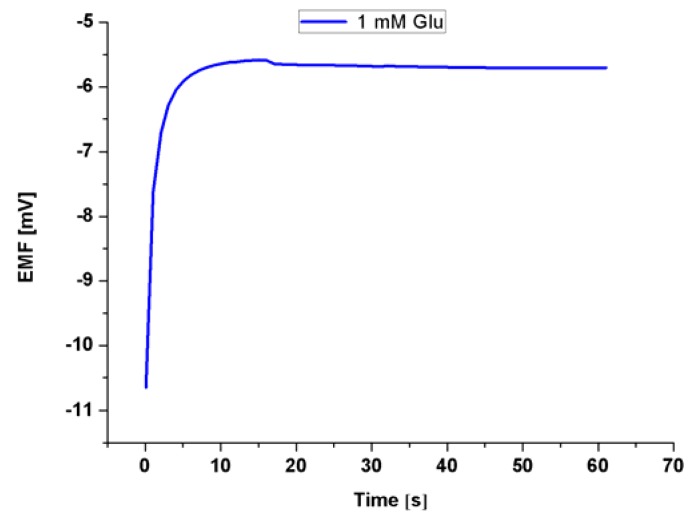
Response time of less than 10 s measured in 1 mM concentration of Glu.

**Figure 9:**
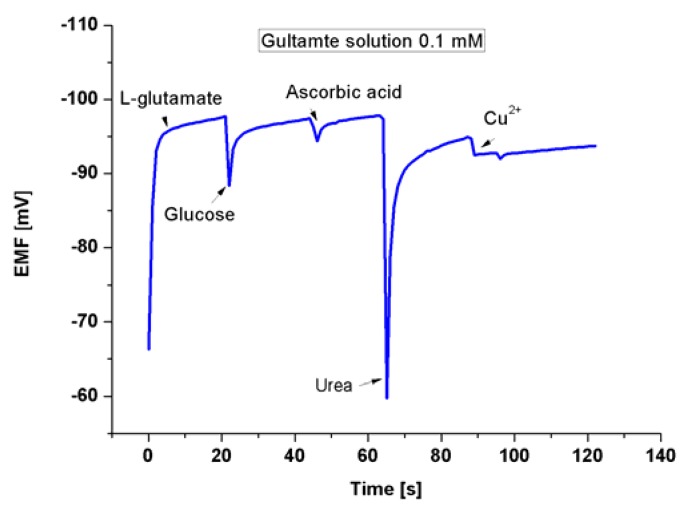
The selective response of the fabricated Glu biosensor in the presence of common interferents at concentrations of 100 µL of 100 mM, glucose, ascorbic acid, urea, or copper ion, respectively.

**Figure 10:**
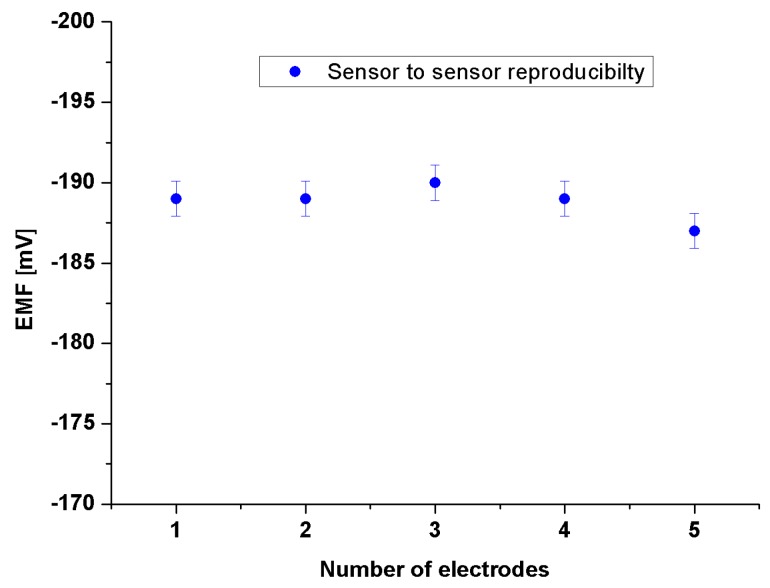
Reproducibility results for the sensor to sensor response in 10 µM concentration of Glu for five sensor electrodes using the same set of conditions and the same functionalized membrane.

The addition of the EG to the growth procedure has altered the electronic band gap, and the carrier concentrations have been modified as indicated by the XPS, and Mott-Schottky analysis respectively. Accordingly, five electrodes based on ZNRs formed in presence of 0.1% w/v of EG and were tested into different concentrations of Glu. A CluOx immobilized ZNRs were used for sensing of Glu molecules using the potentiometric method as has been shown in Scheme 2. The potentiometric response of the proposed Glu biosensor was measured in PBS of pH 7.3 and was carried out for a concentration range of 1 × 10^−6^ M to 1 × 10^−3^ M. The ZNRs that grown without the addition of EG in the above concentrations present no significant response towards Glu molecules with a sensitivity of 29.90 mV/decade (see Figure 7a). Whereas, the GluOx/ZNRs/Au electrodes of ZNRs formed in presence of 0.1% w/v of EG showed a superior sensitivity and a wider range of detection for the Glu concentration with a sensitivity of 91.15 mV/decade (see Figure 7b), and the lower limit of detection was found to be 0.05 × 10^−6^ M. In general the sensing mechanism of a metal oxide based sensor such as a ZnO relies on a change in electrical conductivity due to the interaction process between the surface complexes, such as O^−^, O_2_^−^, H^+^, and OH^−^ reactive chemical species, and the molecules to be detected. The observed wide range of detection and a good sensitivity of the presented biosensor are attributed to the better electro-catalytic properties as a direct result to the doping effect in the ZNRs that is dictated by the used EG along with the high surface area-to-volume ratio [26]. The presented Glu biosensor has showed a fast response time of less than 10 s. The fast response time is shown in Figure 8 and could be assigned to an enhanced electro-catalytic surface activity of the modified ZNRs for a rapid oxidation of the Glu molecules during the measurement. Moreover, the selectivity has been also examined (Figure 9). Upon the addition of 100 µL of 100 mM glucose, ascorbic acid urea, and Cu^2+^ ion respectively to 0.1 mM Glu solution. The results indicate that; such interference is not significantly affecting the Glu signal intensity even with a very sharp signal of urea. This urea signal can be explained according to previously reported work [44]. During the interaction of the urea with a water molecule, then two products are released in the reaction vessel including ammonia and carbon dioxide. The ammonia and α-oxoglutarate are converted to glutamate in a reaction catalyzed by GluOx. Mostly the specificity may be attributed to the existence of GluOx [45]. In order to study the reproducibility the proposed sensor, we independently synthesized five sensor electrodes using the same set of conditions and functionalized membrane. Finally, we measured the reproducibility in 0.01 mM concentration of Glu as is shown in Figure 10. No observable deviations were recorded and it can be inferred that, the response of each biosensor differ from another biosensor electrode by a relative standard deviation of less than 5%. We can infer that, the proposed Glu biosensor developed with EG doped ZNRs works well under the normal conditions of blood serum.

## 4. Conclusions

We have demonstrated the use of EG as a good dopant source that could serve as hydrogen shallow donor source. The addition of EG has altered the electronic gap as indicated by the XPS along with the removal of the OH surface contamination. Furthermore, a slight increase in the carrier concentrations ∼10^20^ cm^−3^ for the ZNRs formed with 0.1% (w/v) of EG was extracted using the Mott-Schottky analysis at the ZNRs/electrolyte interface. The Mott-Schottky analysis also showed that the capacitance dependence on the EG was found to be nearly constant for 0.05 and 0.15% (w/v) of EG due to the doping limitation. The selectivity for Glu was achieved by adding GluOx solution to BSA enzyme stabilizer and glutaraldehyde as a cross linker. Compared to biosensor developed with pristine ZNRs a wider range of detection from 10^−6^ M and up to 10^−3^ M was achieved for EG doped ZNRs. This range of detections would be suitable for Glu concentration in human body and for evaluating the quality of foods. Moreover, the sensor sensitivity was as high as 91.15 mV/decade and a response time of about 10 s. These sensing properties are attributed to the electro-catalytic properties which was triggered by the EG addition to the growth procedure along with high surface-to-volume ratio of the ZNRs. The findings in this paper indicate the importance of the use of controlled nanostructures doping for developing efficient functional materials.
